# Validation of Simple Sequence Length Polymorphism Regions of Commonly Used Mouse Strains for Marker Assisted Speed Congenics Screening

**DOI:** 10.1155/2015/735845

**Published:** 2015-02-28

**Authors:** Channabasavaiah B. Gurumurthy, Poonam S. Joshi, Scott G. Kurz, Masato Ohtsuka, Rolen M. Quadros, Donald W. Harms, K. C. Kent Lloyd

**Affiliations:** ^1^Mouse Genome Engineering Core Facility, Department of Genetics, Cell Biology and Anatomy, University of Nebraska Medical Center, Omaha, NE 68198-5915, USA; ^2^Department of Molecular Life Science, Division of Basic Medical Science and Molecular Medicine, Tokai University School of Medicine, 143 Shimokasuya, Isehara, Kanagawa 259-1193, Japan; ^3^Mouse Biology Program, University of California, Davis, 2795 2nd Street, Davis, CA 95618, USA

## Abstract

Marker assisted speed congenics technique is commonly used to facilitate backcrossing of mouse strains in nearly half the time it normally takes otherwise. Traditionally, the technique is performed by analyzing PCR amplified regions of simple sequence length polymorphism (SSLP) markers between the recipient and donor strains: offspring with the highest number of markers showing the recipient genome across all chromosomes is chosen for the next generation. Although there are well-defined panels of SSLP makers established between certain pairs of mice strains, they are incomplete for most strains. The availability of well-established marker sets for speed congenic screens would enable the scientific community to transfer mutations across strain backgrounds. In this study, we tested the suitability of over 400 SSLP marker sets among 10 mouse strains commonly used for generating genetically engineered models. The panel of markers presented here can readily identify the specified strains and will be quite useful in marker assisted speed congenic screens. Moreover, unlike newer single nucleotide polymorphism (SNP) array methods which require sophisticated equipment, the SSLP markers panel described here only uses PCR and agarose gel electrophoresis of amplified products; therefore it can be performed in most research laboratories.

## 1. Introduction

In recent years, there has been a steady increase in the creation and use of genetically engineered mutant mice for use in biomedical research. Frequently, the genetic background of such mice allows only specific experiments and the mutations have to be transferred to different genetic background(s) to facilitate other kinds of experiments. There are many examples where genetic background is shown to influence the phenotype of a transgenic or knockout mouse line [1–12]. Traditionally however, mutant mice are generated using strains that have shown exceptional performance in terms of their suitability for production of transgenic or knockout mice lines. For example, FVB strain and BDF1 strain mice are most commonly used for transgenic mice production [13] and ES (embryonic stem) cells derived from 129 inbred strains are commonly used for knockout mice production. For technical reasons, chimeras developed in knockout mice generation will carry a mixed genetic background (e.g., 129 and B6) adding further complexity to the analysis [14]. Furthermore, ES cells derived from C57BL6/N inbred strain have been used in mouse genetic resources such as KOMP (Knock-Out Mouse Project) and EUCOMM (European Conditional Mouse Mutagenesis) [15]. Also, particular strains of mice seem to have better suitability for a specific research purpose. In addition, mixed background strains between C57BL/6J and DBA/2J were used for ENU mutagenesis projects because the cross between two different inbred strains (one strain (male) was used for ENU exposure and mated with another strain (female)) is useful for mapping and positional cloning of the mutated gene using genetic polymorphisms existing between them [16, 17]. The main disadvantage of a given mutation under a particular strain background is that the strain background may limit its use for a specific research purpose. In such a situation the mutation is needed to be transferred into a strain background of choice through a process called backcrossing.

Backcrossing involves about 10 generations of successive breeding into a recipient strain of choice to achieve 99.9% congenic (genetic composition) for that strain (http://www.informatics.jax.org/silverbook/). This painstaking process consumes about 2.5 to 3 years of time, a fact that often limits its feasibility and usefulness given the pace of scientific research. In some cases, studies are published with animals after only 5 generations of backcrossing, in an attempt to compensate the time required and the need to obtain some results in the new strain [18]. A technique called “marker assisted speed congenic” used for over a decade helps in achieving congenic strain in 5 or less, unlike the usual 10 generations required in traditional backcrossing [19, 20]. The small sequence differences between mouse strains called “microsatellite markers” served as useful tools in detecting the chromosome regions of origin in the offspring when two inbred strains of mice are bred together. To use in these assays, many microsatellite markers have been identified and characterized by various researchers between the donor and recipient strains of their choice ([21] and [22, page 6] and [23–30]). However, the information about the markers that can differentiate between commonly used strains for transgenic and knockout mice generation is not tested sufficiently and is not available readily. In this study, we tested 423 markers, ~10 to 30 per chromosome, using the genomic DNAs from 10 commonly used mice strains—particularly the strains used in transgenic and knockout research. We evaluated the markers that could be used in the agarose gel electrophoresis method which is a simple technique commonly used in most molecular biology laboratories these days. The data presented here will serve as a valuable tool for various investigators in choosing the markers useful for their speed congenic breeding.

## 2. Materials and Methods

### 2.1. Selection of Oligonucleotide Primers

The primers were chosen based on the following criteria: (1) evaluation and establishment of at least 6 markers per chromosome, (2) distance between the adjacent markers kept as minimum as 10 to 15 centimorgans (cM), and (3) polymorphic bands appreciable when analyzed in a 4% agarose gel electrophoresis and resolved by electrophoresis distance of up to 10 centimeters from the loading wells. All markers were chosen from the Mouse Genome Informatics (MGI) database links (http://www.informatics.jax.org/searches/probe_report.cgi?_Refs_key=22816 and ftp://ftp.informatics.jax.org/pub/datasets/index.html (numbers 7 and 12 in the list)).

### 2.2. Mice Strains

The mice were purchased from Charles River Laboratories or The Jackson Laboratory. The strains, the rationale for including these strains in the panel, and the vendors are listed in Table 1.

### 2.3. DNA Extraction, PCR Amplification, and Agarose Gel Electrophoresis

The DNA samples were extracted from the tail pieces of about 3–5 mm length using Gentra Puregene Tissue Kit (Cat. # 158622). Twenty ul PCR reactions were set up using 1X reaction buffer containing 20 mM Tris pH 8.4, 50 mM KCl, 3 mM MgCl_2_, and 1 unit of Taq DNA polymerase (New England Biolabs, Cat. # M0273) under the following conditions: one cycle of 95°C –2 min followed by 35 cycles of 95°C –30 sec, 55°C – 30 sec, and 72°C–60 sec and one cycle of 72°C – 5 minutes, followed by a holding temperature at 4°C until the samples were removed from the machine. Fifteen ul of PCR products was resolved using 4% agarose gels for 120 to 150 minutes at 200-constant-volt electric current. The agarose was purchased from Phenix Research Products (Item Number RBA-500: Molecular Biology Grade) and the gels were prepared on 0.5X TAE buffer diluted from a stock of 50X (Fisher Scientific, Cat. # BP1332-20). The gels contained ethidium bromide dye (0.5 *μ*g/mL) to aid the visualization of PCR bands. Each panel of gel included one or more lanes of 100-base-pair molecular weight marker (New England Biolabs, Cat. # N3231) to assess the PCR product sizes. The bromophenol blue dye-front was allowed to run for up to 10 centimeters from the wells and the gels were imaged using BioRad Gel Doc XR system. Wherever necessary, the gels were run longer to resolve the bands.

### 2.4. Analysis and Interpretation of Polymorphic PCR Bands

**Figure 1:**
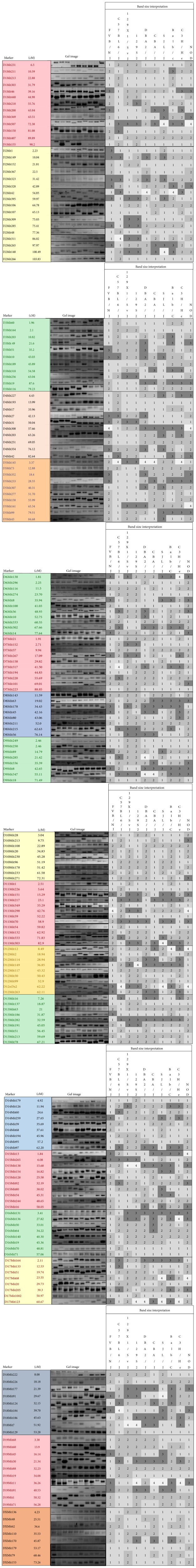
Agarose gel images of PCR bands and their interpretation. Bands were assigned numbers 1, 2, 3, or 4 to indicate their sizes compared to the rest of the bands in that set. Number 1 was assigned to the smallest sized polymorphic band. 2, 3, and 4 represent the successive bigger sized bands.

The cropped images were imported into an Excel file for analysis and interpretation. Band sizes were assigned numbers 1, 2, 3, or 4 to indicate their sizes compared to the rest of the bands in that set. Number 1 was assigned to the smallest sized polymorphic band, 2 to the next biggest in the group, and so on. The Excel file along with the gel images was converted into a  .pdf file for generating Figure 1.

## 3. Results and Discussion

Mutant mice created using transgenic and knockout techniques are available under certain specific backgrounds. In order to best use such mutants for a specific research purpose, they routinely need to be bred into other strain backgrounds through successive breeding of about 10 generations. A quicker way to attain highest recipient genome can be achieved by a process called speed congenic breeding in which the polymorphic markers between the recipient and donor strains are screened among offspring in each generation and the offspring with highest recipient genome is chosen as a breeder for the subsequent generation [19–21]. Although there are a few reports describing the marker sets suitable for certain pairs of strains, there are no well-established marker sets available across the most commonly used strains in transgenic and knockout mouse techniques. Our primary objective of this work was to test the suitability of several markers in an agarose gel electrophoresis method, a technique that uses least expensive equipment and reagents and is readily available in most molecular biology laboratories. We sought to test a large number of microsatellite markers (Tables 2 and 3 and S1 in Supplementary Material available online at http://dx.doi.org/10.1155/2015/735845) among 10 most commonly used mouse strains particularly those used in transgenic and knockout mice techniques.

We used Mouse Genome Informatics (MGI) database and short-listed the primers based on the criteria described in Section 2. MGI database lists several of the microsatellite markers identified in various inbred strains. Although the database is extensive, it is difficult to choose marker sets for speed congenic screening because it lacks the critical information such as the sizes of PCR products of various markers in different inbred strains and if the differences among strains can be identified using conventional agarose gel electrophoresis.

As per the information available on MGI database, predicted PCR band size differences among strains for some markers ranged from as few as a few base pairs to over 100 base pairs. Selection criteria for markers and the mice strain are described in Section 2. Taking C57BL6J and 129X1 strains as a comparison pair, for example, we chose markers with differences of 30 bp and above (as per MGI database), a range that can be easily resolved using about 2% agarose gels. We aimed to choose markers in this range for all the chromosomal locations with an interval of 5 to 15 cM. However, we were unable to find suitable markers in some regions with this criterion. In such locations, we tested markers with as less as 8 to 12 bp size difference. Such small differences can be best resolved using polyacrylamide gel electrophoresis (PAGE). However, in a typical speed congenic screening that involves analysis of about 100 markers for each sample, applying PAGE method for screening becomes quite laborious. We used 4% agarose gels to resolve makers even with very small size differences; this seemed to be sufficient to resolve the bands when they were run about 8 to 10 centimeters from the loading well. In order to keep the assay conditions uniform we used 4% agarose gel for all the markers tested.

Table 2 shows the list of the markers tested and found polymorphic in at least one of the 10 strains analyzed. We tested a total of 423 markers of which we present useful data for 195 markers. The gel images and interpretation of polymorphisms are tabulated in Figure 1. The gel images indicate that there were readily appreciable size differences for most markers whereas some markers showed very narrow size difference between some strains. In general, sizes of the majority of the markers matched the information on MGI database but there were some discrepancies noticed. The agarose gel electrophoresis using 4% gels could be useful in detecting differences among the markers in different strains.

The data presented in Figure 1 indicate that some markers could readily distinguish several strains from each other. We initially aimed to identify good panel of markers for every 10 cM. Although we found adjacent markers as close as 10 cM in most locations, we failed to achieve this in some regions. We screened several available markers in such regions and were unable to find markers that matched the criteria set forth in our assay. It should be noted that there were markers that differed very minutely among some strains. If such markers are chosen in an actual speed congenic screening, we recommend including samples from both donor and recipient strains and an equimolar mixture of the two in the panel in order to ensure the proper banding pattern of offspring. The gel images and the interpretation of polymorphism provided in Figure 1 will serve as a comprehensive tool for a researcher who wishes to undertake congenic breeding in a pair of strains from the list.

The data from the markers that did not meet our criteria fell into one of the following categories: it (1) yielded the same size bands in all the strains, (2) failed to amplify a product in some strains, (3) amplified multiple PCR products, or (4) did not yield a reliable PCR product in most/all of the strains. Category 1 means that the bands were not resolvable by 4% agarose gels. We presume that such markers would be <6 base pairs different from one another [31] or they do not differ in size at all. Category 2 would probably mean that the primers did not have perfect binding sites in those strains where there was no PCR product. Category 3 makers may need further optimization of PCR conditions. However, one of our aims was to keep the conditions uniform to all the markers in order to keep the assay simple: it makes the assay cumbersome if PCR conditions have to be varied among different marker sets. It should be noted that the category 4 markers (that did not yield a reliable PCR product in most/all of the strains) might have not worked due to technical errors that may have occurred during some steps like primer synthesis or primer reconstitution; we do not rule out the possibility that some of these primers may yield PCR products if they are resynthesized. The markers that did not meet our criteria because of the above reasons are listed in Table 3 and Supplementary Table S1: providing the list of primers and markers that failed in our hands will help researchers to compare the results if they intend to test more markers to expand the streamlined panel presented here.

There is some debate about the minimum number of markers needed per chromosome for identifying the regions between the donor and recipient strains [32]. A study concluded that as few as three markers per chromosome were sufficient to achieve similarly meaningful results as that of over 6 markers per chromosome [33]. Computer simulation [20] indicated that a relatively modest selection effort of 60 evenly spaced markers with 25 cM spacing (corresponding to 3 markers per chromosome), 16 males per generation, would typically reduce unlinked donor genome contamination to below 1% by four backcross generations (N5). We conclude that the list presented here can serve to choose panels of markers for most two-strain combinations with at least 3 to 4 markers per chromosome (with exception of a very few combinations). Further studies that compare smaller and larger panels of markers in the same set of samples for marker assisted speed congenics are needed to address this question unequivocally.

In recent years there have been advancements in the approaches used for speed congenics. These methods include (i) use of fluorescently labeled primers to amplify SSLP markers followed by resolving the products in sequencing gels [29] and (ii) microarray chips of SNPs [29, 34–36]. With the advent of newer methods particularly those that use SNP based marker analysis, a very high number of markers per chromosome can be screened simultaneously that increases the resolution severalfold compared to the conventional SSLP based markers analysis. Although there are some computer simulation studies for assessing the efficiency of speed congenic screening in general [32] and algorithm based reports to compare SNP and SSLP based speed congenic screens [37] there are no systematic studies to compare the two methods to assess the efficiency and cost effectiveness of each method. Here, we compare the SNP and SSLP based approaches in terms of their adoptability and feasibility to most laboratory settings including their cost. The hands-on-time in performing gel based assays has been reduced greatly by newer methods that use SNP arrays. However these methods have some limitations compared to traditional agarose gel electrophoresis (AGE) based SSLP marker analysis. (1) The newer methods are expensive in terms of the initial investment in reagents and/or operational costs compared to SSLP-AGE method. On the contrary, basic requirements needed for AGE based systems are readily available in any molecular biology laboratory and the only additional investment needed will be to synthesize the required oligonucleotides. (2) Subset of markers to be analyzed in the subsequent generations cannot be skipped from the panel unlike in the AGE based method where the number of markers to be analyzed will become significantly reduced in successive generations and so also the overall cost of the assay. Other advantages of AGE based SSLP marker analysis over these newer methods are as follows: (i) the equipment needed to perform the assay is readily available in most laboratories and (ii) it can be routinely performed by many researchers and technicians without the need of special training as needed for SNP based approaches.

Considering the highest resolution that is possible with the SNP based method, it can be regarded as the superior method of all. However, the microchips that are currently available are expensive and the cost for analyzing each mouse DNA sample runs to about US $100 to $150. Assuming that about 15 mouse DNA samples per generation for 5 generations are analyzed, a typical speed congenic project would cost about $15,000 to $22,500. On the other hand, when using SSLP-AGE approach, since marker analysis is done manually, the markers that were fixed in the previous generation can be skipped in the successive generations; this makes the SSLP-AGE based method cost effective compared to SNP based method. It is estimated that a typical SSLP based speed congenic screen that employs analysis of 15 samples per generation for 5 generations using 5 markers per chromosome would need about 2000 to 2300 PCR reactions. At the rate of $2 to $2.5 per reaction (cost analysis done in our laboratory) it will cost about $4000 to $5750 for one full speed congenic project. Furthermore, SSLP-AGE method can be performed in any simple molecular biology labs compared to SNP method that requires expensive equipment.

## 4. Conclusions

Although some information about microsatellite marker differences between the commonly used inbred mouse strains was available, there was no systematic study to validate a large panel of markers for SSLP-AGE based speed congenic screening. The panel of marker sets validated and presented in this study serves as a ready reference for researchers who wish to perform cost-effective speed congenic screening in a pair of strains from the panel. The assay can be performed in any standard molecular biology lab. The data in this report is available at ftp://ftp.informatics.jax.org/pub/datasets/index.html#Guru.

## Supplementary Material

Providing the list of primers and markers that failed in our hands will help researchers to compare the results if they intend to test more markers to expand the streamlined panel presented here.

## Figures and Tables

**Table 1:** 

Strain	Vendor, stock number	Rationale
C57BL/6J	JaxMice, 000664	Commonly used strain in biomedical research

FVB/NJ	JaxMice, 001800	Commonly used strain for transgenic mice generation

129 X1/SvJ	JaxMice, 000691	Strains from which highly successful germ line transmission competent ES cells were derived
129S2	Charles River, 476	

DBA2/J	JaxMice, 000671	Together with C57BL6, these strains are used as hybrid strains for transgenic mouse production
CBA/J	JaxMice, 000656	
SJL/J	JaxMice, 000686	

Balb/c	Charles River, 028	Is among the top 2-3 most widely used inbred strains

C3H/HeJ	Charles River 025	Used in a variety of research areas including cancer, infectious disease, and cardiovascular biology
NOD	Charles River	

**Table 2:** Primer sequences used for PCR amplification of SSLP markers.

Marker	cM	Primer F	Primer R	Product size	Lab code
D1Mit231	6.5	ACCCACAATTGCCTGTGG	GTCTTTGCAAGCCACCAAAT	267	1-1
D1Mit211	10.59	GTTATTCATCAAAATACAGATGGCC	TCTGCTGCTAAGTAGAATGAATGC	135	1-2B
D1Mit213	22.88	TTCTTAGAAGTGATAAAAGTTTCAGCA	AAAATTCCAGAATTCTCACTACGG	108	1-2
D1Mit303	31.79	GGTTTCTATTTCGGTTCTCGG	TCTGTGCTGCAAAACAGAGG	128	1-3
D1Mit46	39.16	AGTCAGTCAGGGCTACATGATG	CACGGGTGCTCTATTTGGAA	253	1-4
D1Mit440	44.98	TCCACACAAGGTGTCCTCTG	GCTCAGGTGACCTCCAAAAC	114	1-5B
D1Mit218	55.76	TGCAAATGTTACTTTAGTCTCTAGTGG	AGTTTTGGTGAGTGATCTTATCCC	145	1-218
D1Mit369	63.51	ACTTGTTTGTTGCTGAGGTTCA	GCTTATGAACCCACCCTCAA	141	1-5Q
D1Mit200	63.84	GCCATGTTCATGTACATAGGTAGG	ATGGATGGATGGTTTTCCTG	199	1-200
D1Mit507	72.38	GTTGGAAAGACTGTTTACAATTCG	GTTCCAGCCTTTGCCCTTAC	115	1-6
D1Mit150	81.08	CTGGTGTCCACACACAGGTC	TGATTGCAATATACCAGGTTTCC	138	1-150
D1Mit407	89.89	GAGAACAACCAGCCACCAAT	ATATTTGCTTTGAAGTTACTTTGTGTG	119	1-407
D1Mit155	98.2	ATGCATGCATGCACACGT	ACCGTGAAATGTTCACCCAT	252	1-7

D2Mit1	2.23	CTTTTTCGTATGTGGTGGGG	AACATTGGGCCTCTATGCAC	123	2-1
D2Mit149	10.04	ATATCATATAGTAGAGAAAGCGTGCTG	TCATTAGACTTGGAAAAAAGTTTGC	199	2-1P
D2Mit152	21.81	CACAGATCTTGTAAGACCACGTG	TGCCATGAGTGTGGGACTAA	109	2-2
D2Mit367	22.5	GCCTGTGCTAAAAAAGAGGTG	GCCCTGAGAACTACCCTCCT	149	2-367
D2Mit323	31.42	AGAATCCTAAGTGGTGGTTAGAGG	ACCCAAAGTTGTCTTTAAGTACACA	125	2-2P
D2Mit328	42.89	CTTTCAATGTTCCGGCATG	AAGACTTGCTTTCATTAGACCACA	235	2-3
D2Mit42	54.85	ATTACTGGGCAGGAACATTTG	GCCAAACTTCCAGACTCCTC	132	2-3B
D2Mit395	59.97	AGGTCAGCCTGGACTATATGG	AGCATCCATGGGATAATGGT	125	2-4
D2Mit106	64.78	GAGGGTTGCCAAAGAGACTG	CACCTCAGGGGAACATTGTG	150	2-4P
D2Mit107	65.13	GGGAGTGAAGCCAGCATAAG	AACTGACTGAGTTTCAAAGTGCC	119	2-107
D2Mit309	75.03	ACAAATGCCACTCTCACATCC	TATTTCTCAGAGTCACTAGGAGTGATG	119	2-309
D2Mit285	75.41	TCAATCCCTGTCTGTGGTAGG	TATGACACTTACAAGGTTTTTGGTG	141	2-5
D2Mit48	77.36	GCTCTGCAGAAGATGCTGC	GCTGAGACGCAGAGTCGC	130	2-5B
D2Mit311	86.02	ACAGGCAGCCTTCCCTTC	TCTGTCCCGCTTCTGTTTCT	126	2-5D
D2Mit265	97.97	AATAATAATCAAGGTTGTCATTGAACC	TAGTCAAAATTCTTTTGTGTGTTGC	105	2-6P
D2Mit266	103.83	GGATCTATGCTCCATTTTAATTGC	TCATCTTCTGGTTTCAACATGG	127	2-6
D2Mit148	100.49	GTTCTCTGATCTACGGGCATG	TTCACTTCTACAAGTTCTACAAGTTCC	115	2-148

D3Mit60	1.96	GACATCCTGGGCAACATTG	GGTGTTGTTTGCTGTTGCTG	170	3-1
D3Mit164	2.01	GCTCCTGGGAAAGGAAGAAT	GATACTTGGGGTTGTGCATACA	135	3-164
D3Mit203	10.82	CTGAATCCTTATGTCCACTGAGG	GGGCACCTGCATTCATGT	150	3-2
D3Mit51	35.2	GGCACTGATAGCAGGCCTAG	TCTCTTCTGGTATTTCCTTCCG	248	3-3
D3Mit 49	39.02	CTTTTCTCGCCCCACTTTC	TCCTTTTAGTTTTTGATCCTCTGG	132	3-49
D3Mit10 F	43.03	CTGGCTTGGTGGAAGTCCT	CCTAAGCCAGCTACCACCAC	142	3 10
D3Mit189	43.89	GTTACCACCCAGAGAAAAGGC	TACTCCTCGCTGCTTCCCTA	133	3-189
D3Mit318	54.58	CTCATTCCTTCTGAGCAATGG	TATGGGATATGCTTTTCATAAAAGG	148	3-318
D3Mit256	63.04	TACATTGCTTTTTGCTTTGAGTG	GTCGAATGTTATCAGAATTTGCA	125	3-4
D3Mit116	79.23	TCACTGCCCATCTTTGTAACC	CCCAGAGACCCGGAATAGAA	259	3-116
D3Mit19	87.6	CAGCCAGAGAGGAGCTGTCT	GAACATTGGGGTGTTTGCTT	159	3-5

D4Mit227	4.43	CTCAGACATGATTTTTTCCAAGG	GCAGTTAAACTGTACTTTCTGTAAACA	181	4-1
D4Mit193	13.99	TATTTTAATTTTAGCCCATCAGGG	AAAGACATACAATTGATCCACAGG	136	4-1B
D4Mit17	33.96	TGGCCAACCTCTGTGCTTCC	ACAGTTGTCCTCTGACATCC	147	4-2
D4Mit27	42.13	GCACGGTAGTTTTTCCAGGA	TGGTGGGCAGGCAATAGT	150	4-2B
D4Mit31	50.04	ACGAGTTGTCCTCTGATCAACA	AGCCAGAGCAAACACCAACT	121	4-2C
D4Mit308	57.66	TATGGATCCACTCTCCAGAAA	CAAAGTCTCCTCCAAGGCTG	88	4-3
D4Mit203	63.26	GAATTCTTCCTGGGCCTTTC	CAAGAGCCCAGGTGTGGTAT	105	4-3B
D4Mit251	69.05	AAAAATCGTTCTTTGACTTCTACATG	TTTAAAAGGGTTTCTTTATCCTGTG	114	4-4
D4Mit354	76.12	TTGATCTGTCGTGGATTCCA	AGACAGACACATAGACACAGACATAGG	111	4-4B
D4Mit42	82.64	CATGTTTGCCACCCTGAAAC	CCTCACTTAGGCAGGTGACTC	100	4-5

D5Mit145	3.37	TATCAGCAATACAGACTCAGTAGGC	TGCCCCTTAAATTCATGGTC	150	5-1
D5Mit73	12.88	GTTTGAGAGGTCCTGAAAGCA	TTTCCATTTACATACATTTGTGCA	113	5-1B
D5Mit352	18.4	CCCAGAGCCCACATCAAG	TAGGTGGGTGTGTCTCTCCC	110	5-2
D5Mit233	28.55	TCCCCTCTGATCTCCTCAGA	CCTCCTAGAATACAATTCAATGTGG	147	5-3
D5Mit307	40.31	AGTGGCATAACTTCATTCAATAATG	GGAATCAAGTTGTTTTTTAAATTTACC	120	5-3P
D5Mit277	51.7	GTGTGTTTGTGCATGGGTATG	ACCATCGGGAAAAAATGTAGC	123	5-4B
D5Mit161	65.34	CACACGCATAGTCTTGTGGG	GCATGTTCAACTGTGCTTTCA	119	5-5B
D5Mit99	79.51	CAGAAAAGAGAAAACGGAGGG	TTCCTGCTGCCTGAAGTTTT	200	5-5C

D6Mit138	1.81	GCTCTTATTAATGAAGAAGAAGGAGG	CAAAGAAAGCATTTCAAGACTGC	135	6-1
D6Mit296	2.25	TCGGGCATCTTTATTTTTGC	TAGTGCAGCACACCCCCT	100	6-296
D6Mit116	11.5	ACATTTCTTTGTGAGGTTCCTTG	CAGGTTTTTTGAAAGACACTCTTG	122	6-1B
D6Mit274	23.7	GCAATGCCAAAATGTTCAAA	TCCTTCTCCATTTACACTTACAACA	113	6-1D
D6Mit8	35.94	TGCACAGCAGCTCATTCTCT	GGAAGGAAGGAGTGGGGTAG	163	6-2B
D6Mit100	41.03	CTTGAGTAGGTCTCAGTGCGG	CACATGCACACACAGAAGCA	84	6-3
D6Mit36	48.93	ACCATCTGCATGGACTCACA	GTTGAAGAGGACGACCAAGTG	196	6-4
D6Mit10	52.75	TCAGAGGAACAAAGCAGCAT	CCTGTGGCTAACAGGTAAAA	200	6 10
D6Mit333	60.55	TCCTCACTACAATTCATCTATTACTGC	TGCTTCTGGTATAGGCAGTTAGG	122	6-333
D6Mit302	67.66	AATGACCCTGGTTAGTGTCAGG	GAATTCCATTCGAGGGGC	113	6-4C
D6Mit14	77.64	ATGCAGAAACATGAGTGGGG	CACAAGGCCTGATGACCTCT	157	6-5

D7Mit21	1.91	GGGTTGAACCTTACAGGGGT	ATCAAACCAGCCCAAGTGAC	192	7-0A
D7Mit152	2.71	GCCTAGCACACGCCAAAG	CCTTGTGCATGGTTGCTATG	129	7-152
D7Mit57	9.94	TTCCCTCTAGAACTCTGACCTCC	AGTTCAGAGCCGAGACTAGGC	148	7.-1
D7Mit267	17.09	CTCTTTCTGTTACATGGTTAGATTTCC	AAAGACAGTTGAAGTTGACTTCTGG	192	7-2
D7Mit158	29.82	CTTCATCTGAGCCTGGGAAG	ACTGTAGACCCATGTTCTGATTAGG	149	7-158
D7Mit317	41.5	ATGTCTCCTTGACATTGGGC	TCTTGTAATCTCACATCTAAGTGTGTG	102	7-2D
D7Mit194	44.83	GTGCAACACACAGAAAAGTTCC	AAAGGCTCACAACGGACTGT	146	7-194
D7Mit220	55.69	AAGCATGCAAGCACACTCAC	ATGCACACAGGCAGTCACTC	135	7-3A
D7Mit101	69.01	TACAGTGTGAACATGTAGGGGTG	TCCCAACATGGATGTGCTAA	111	7-4
D7Mit223	88.85	ATGCACATGAGTGTGTGTATGC	TCCTGTGTCTGACGCTCATC	106	7-5

D8Mit143	11.59	AGCCTGAGGTTATGTTTTTTGC	GGCCCTCAGGTTTTCTCTCT	279	8-143
D8Mit178	34.43	AAAATCAACTGTTTACATTTGAGCC	AGAGCACGCAGTGTGTATGC	148	8-2
D8Mit45	42.16	GAACAGGACCAATAAAATGAAAGC	CTACCTTACCAAACTTCCCGG	121	8-45
D8Mit80	43.06	TGCATTTGTCAGGGCTCTC	ATGACACATGAGCCTCCACA	107	8-080
D8Mit211	52	CAGAACACTGTCCTGAAAAGTCC	TACCCACAAACCTGTATTTAAATTAA	149	8-4
D8Mit215	62.63	AATACACAAGGTTGGCCTCA	ATGTGTGGATATTCATGTGCTC	178	8-5
D8Mit56	76.14	ACACTCAGAGACCATGAGTACACC	GAGTTCACTACCCACAAGTCTCC	162	8-56

D9Mit250	2.46	CCCAAAAACCTATTTGCAGTG	GTGACATGATTCCTTCAGTCTTACC	123	9-1
D9Mit249	2.46	AAGCCCTCTTAGAAGTAGTGTGTATG	AGCCATGAACTAACTTACATGTATCA	125	9-249
D9Mit89	14.79	CACATACAAGGATATACATACACAGGC	TCACAGGAGGTGGCAGAAAT	145	9-89
D9Mit285	21.42	CAAATACATTGCTGATTATATCAGAGA	GGACTCTAGATCTCATCAGGGA	125	9-2
D9Mit336	35.39	AAGTGGTTCACAGAAATGTATACAGG	TTTTCTTTCTGTGGTAAAGGGG	122	9-3
D9Mit8	42.65	GATGAAGACAATAAAGAACCTTAAA	AAGAGCTAACCCATTGCTGC	183	9-3B
D9Mit347	55.11	CCTCCACATGTGCACTGCT	CTGTCCATCTATCATCTATCTGTCTG	122	9-4
D9Mit18	71.49	TCACTGTAGCCCAGAGCAGT	CCTGTTGTCAACACCTGATG	180	9-5

D10Mit28	3.04	CCTCCTGTATGTGTATTTAAAGCA	CTGCCCATCTGACCCTGATA	147	10-1A
D10Mit213	9.75	CTCCTCCTACTGATTGTCCCC	GGGACAAACTTTTAAAAATTGCA	150	10-2
D10Mit108	22.89	TGCCTGTAACCTGCATACCC	GTTTAACACCCAGGACTATACATGG	142	10-108
D10Mit20	34.83	CACCCTCACACAGATATGCG	GCATTGGGAAGTCCATGAGT	234	10-3
D10Mit230	45.28	AGATAGCCTAGGGGGTGCAT	ATCAGTTTCCAATCGCTGCT	115	10-4
D10Mit96	51.19	CTTCTTTGAAGTTAGATGCAGCC	TACGGAGAAGGGAACACCTG	150	10-096
D10Mit178	51.42	ATTGTCAAATATCTTCCTCAGTTGC	TTATTCCTAGGCAGTCTGTCTGG	133	10-4B
D10Mit233	61.58	GTGCTTTATATTGGAGATCATCACA	GTCCCGAATTTCACATACATAGC	130	10-5
D10Mit271	72.31	ACAACCAAAGGTCTTTGTAGAAGA	AATATATAGGCACACCTTAATAGCCA	117	10-6

D11Mit1	2.51	GGGTCTCTGAAGGCTTTGTG	TGAATACAGAAGCCACGGTG	153	11-1P
D11Mit226	5.64	AGGTGAACTCTTTTGAAGTTTGTG	AAAGGAGTGACTGAGAAAGACACC	139	11-226
D11Mit151	15.29	TGGGAATTCTGGGAGTTCTG	GTTGGTCTGTTATGAAGACCAGG	140	11-1Q
D11Mit217	23.1	ACTGGAAAATATGTTTTAAACCTCTG	AAATGGGATTCTGCAAAAACC	135	11-217
D11Mit349	33.29	AGTATCAGAAGATCCAGTTGGAGG	GTAGAAAAAGATACCCAGTGTCAGC	118	11-349
D11Mit298	42.76	AAACAAACAAAAATGCACCTCA	GTACCACCATGCCTAGCCTC	199	11-298
D11Mit39	52.22	TTTCATGACCCCTAATTTCCC	GTGGGTGTGCCTGTCAATC	155	11-39
D11Mit70	58.9	GGAAGTAGCTATGGAGGTGGC	TCTGACCCAGAGCTCAAATACA	140	11-70
D11Mit54	59.82	AGGCTGGTGGCTAGTGTCC	AAGTCTTGCGCTGCATCTTT	144	11-4A
D11Mit132	62.92	GGTCAGAGGACAATCTTACATGC	GTTCCAAGACAATGAGAGACCC	117	11-4B
D11Mit333	71.83	CATGTGGTTATTTTCTAGCCCC	AGGCATCAATAACTATTTTTCAGTG	125	11-5
D11Mit303	82.9	TCAATCTCTCAAGTTTTTCCAGC	GACAACGTTGACCTCCACG	106	11-6

D12Mit12	8.49	TTCAATGCCTTCTGGCTTCT	GATTACCGGGTGTGTGACCT	145	12-1
D12Mit2	18.94	ACACAGGCTAAAACATGGGC	GCATCTGTATTCCACAGGCA	134	12-2
D12Mit114	28.94	TTGACCTTGAACTTGTGACCC	GTTTTCTCCAAATCACTGTCACC	144	12-3
D12Mit149	36.85	CATGGCACACATACATACATGTG	AACATAGCAATGGTATATAGGTATGGG	132	12-3B
D12Mit117	43.32	AATTGAGGAACTTAGAAGAAAAGCC	CCTCTGGCCACCATACATG	127	12-117
D12Mit30	50.43	TATGTGACTGCAATCCCAGC	ATGAACACATCATGCCCAGA	107	12-30
D12Mit99	52.9	CTTACAGAAAATGAAAACCAAAACA	CCTCTGCTTTAGAGGCAAACG	151	12-099
D12Mit263	62.11	TCAGATCTCAGCAGATAAATACTTGG	TCCCCTGGAGCATATTTGAC	113	12-6
D12nDs2	62.22	ACATGGTAATTTATGGGCAA	CTGGATACCTGCAATAGTAGA	195	12-5

D13Mit16	7.26	CCAGCTGAAGGCTTACTCGT	AAAGTTAGAATCAGCCATTCAAGG	207	13-1
D13Mit137	18.87	GAATCAGAGAACCTGGCTGTG	TCTAAAAGAGAGAAAATTGGGGC	131	13-137
D13Mit63	21	GAGATGGAAGGAAGAGATGGC	CAAATGCATGTATCCGTATGTG	139	13-63
D13Mit186	31.87	GAAAGCCCTAGGGGAAGATG	TGCAGTTTCTAAGGTTAAAACTAAAGC	149	13-3
D13Mit282	32.59	TCGCACTTCCTATACAGTTATAAGAG	GGACAGAAAGCATGCAGAGG	125	13-282
D13Mit191	45.05	GCAAATTAGAGAATCATGCATCC	TGGAGAATGCTAAAAGCATGG	119	13-3B
D13Mit51	56.45	TCCTGCAAAAGTGGAGCC	TGGAAACAAGCTCTTGGAGG	146	13-3P
D13Mit213	59.69	GCCTGAAACTCTACATAAAATACATCC	AGTTTCATTGCTTTAGTTACATTTTCA	149	13-5
D13Mit78	67.21	ACAGCACGGGTTTATCATCC	TATGCCTGCCAGGCTTCTAT	229	13-6

D14Mit179	4.92	CCACTTGCAGCATTGACAAT	CAAACATCTGTGACAATAAAATTTCA	144	14-1P
D14Mit126	11.94	CCTGTCCCACAACACCTTTT	TATACATATGGGTAGCACTGAGTGG	140	14-2
D14Mit60	24.6	AGGCTGCCCATAAAAGGG	GTTTGTGCTAATGTTCTCATCTGG	132	14-3
D14Mit259	27.65	TGGTGTCTCCTTCGGAATTT	TAAATGTAAAAGGTAAAGGCAATGG	125	14-259
D14Mit39	35.69	AAAGAGCAACCCCCAATTCT	ACTTTTACCTGGTCTCCAAAAGC	246	14-39
D14Mit68	37.61	GTGGCATGCACAACCGTATA	CCCTTTTGAGGTGCTTGTTT	153	14-4
D14Mit194	45.96	AATATTCTAAATGAAATCCAATGTGTG	TTAATTGCAAGTAACACAATGAGTAGG	92	14-4B
D14Mit95	57.2	TATTTTTAAGTCAGTATACACATGCGC	TTATCCAAGTGTATTTAAAGAAGAGGC	124	14-5
D14Mit97	62.2	TCAGTCCAAACTCTGTTAATCTTCC	CAGCTCCACATTTTTGCTCA	136	14-6A

D15Mit13	1.84	GGAGACAAAAATGAACTCCTGG	TTGTAAGACAAGCATAGCTCAACA	136	15-13
D15Mit265	6.08	ACATTAGTCAACTATGCTGGTACTCTG	TTCCTCTCTAATGTCAATTGTTTCA	198	15-265
D15Mit138	15.68	TTCAATTCCCTTTTGTCAAATG	CAAGACCCTAGATTCAGTCTACCC	147	15-138
D15Mit154	16.82	AGCACTGGGTACACAAACTGG	ATGAAAGCATGTGTAGTCTTTCTCA	150	15-2
D15Mit128	25.58	CAAGTTCTGCAAAGAATTATTTATGC	CCACTTTGAAGTTTTCTTTCTTAGC	134	15-3
D15Mit92	32.19	AGTCTCTCTCCCCCTTCTCTC	TGCCACAAGCACAATAGTATCC	147	15-3B
D15Mit80	38.02	CATTGAGGGTTTGTAGGTTGG	ACCCCTGCAAGTTGTCTTTG	149	15-4
D15Mit34	45.31	TGGACAACCATTTTGGACAA	CTTTCTGTCAGGCATCACCA	147	15-34
D15Mit244	48.65	TCTACCCTCTGTGGAACATCG	CTTTGTGTCCATACACTAATATCAAGG	116	15-4P
D15Mit16	58.05	AGACTCAGAGGGCAAAATAAAGC	TCGGCTTTTGTCTGTCTGTC	119	15-6

D16Mit131	3.41	TGGTGGTGGTGTTGATGGTA	AAGACCATTTCTAATAAACAACACCC	140	16-1
D16Mit136	27.82	AGATAATTCCCGTGAGAATAAAACC	TTGAGAAGTTTGCCCTATAATGG	129	16-2
D16Mit30	33.01	GTGCACATACATACCACAGCG	TCACTGCAGGGAGGTTCAG	152	16-30
D16Mit64	34.22	TACCATGATCAGTCCAAAGGC	ACTTAAGGTTGTCCTGTGGGG	220	16-3
D16Mit140	40.3	ATAGTTGAAAAACTTGAACATGCG	GAAAAGGTTAATGCTGGTCACC	145	16-3B
D16Mit19	45.36	CAGGCATGTGAACAAAGTGG	GTGACTGATGAATGCCTGACA	121	16-3C
D16Mit70	48.81	GGATCTATATGCTATAGAACCATTCA	GTCATCAATTCCATTTCCTAATATAGA	187	16-4
D16Mit71	57.06	TAGAAAATCTTCAAATAGGATCTGTTC	GAGCATTTCCCTTTTACCTGG	154	16-6A

D17Mit164	2.11	AGGCCCTAACATGTAGCAGG	TATTATTGAGACTGTGGTTGTTGTTG	133	17-1
D17Mit133	12.53	TCTGCTGTGTTCACAGGTGA	GCCCCTGCTAGATCTGACAG	188	17-1C
D17Mit51	19.74	TCTGCCCTGTAACAGGAGCT	CTTCTGGAATCAGAGGATCCC	154	17-2
D17Mit68	23.55	GTCCTGACATCATGCTTTGTG	CTACCGTTTGGAAGGCTGAG	130	17-68
D17Mit20	29.73	AGAACAGGACACCGGACATC	TCATAAGTAGGCACACCAATGC	165	17-3
D17Mit205	39.3	TGTGCATGTATATGTGTGTGAATG	GCTAAGTCAGAAGAGTTCTGTAATGG	223	17-4
D17Mit1002	50.97	TCTGAATGCTGACTTCCATCC	ACACATATGTGTAGTGTATGAATGTGC	138	17-4P
D17Mit123	60.67	CACAAGGAGGGAGCCTGTAG	CACCGTAAGAGTCTAATAATAAGGGG	133	17-6

D18Mit222	8.08	AATCCAAGATTGACATGTGGC	CTTAGATGCCCTGTCTTAAAAAAA	113	18-1
D18Mit226	18.18	CAGGCAGGGTGCATATATTATAA	TATCTGTTTATGTGTGTACATTGTGTG	125	18-1C
D18Mit177	21.39	CTGTAGTTTATCAGTTCACCCTGTG	TGTGCTGTTAAACAAATATCTCTGG	171	18-2
D18Mit91	29.67	TCCACAAATGTTGGCAAAGA	TTTCTGGCCATATTGGAAGC	140	18-3
D18Mit124	32.15	CCCAAATGGGGTGTCTTTTA	CTGCCACACATTTGTGTGTATG	150	18-4
D18Mit184	39.7	CACACATGTGTAGGTAGGTAGGTAGG	CGCACAAGGACTACTGAAACA	172	18-4C
D18Mit186	45.63	AAGTGTTGGGCAAAGGCTAA	CTTTAGTATAGTGTGCATGAGTGTGA	125	18-5
D18Mit7	51.92	ACAGGAGAACGGGAACTCAG	GCCAGAGTGGACCAAGATGA	95	18-5B
D18Mit129	53.28	CCAGCACAGAGGCAGTCAT	TGATTCTTGGGTCCTGAATACA	138	18-5C

D19Mit68	3.38	CCAATACAAATCAGACTCAATAGTCG	AGGGTCTCCCCATCTTCCTA	132	19-1
D19Mit60	13.9	CAACACCTCACTGTTTAGGAACC	GCTGAGGTCAATATTTAGCATGC	136	19-60
D19Mit45	16.14	CCATTCATAAAATGGGCTTAGG	ACCATGAATGTGTTTTGAGGTG	145	19-2A
D19Mit30	21.34	GGTGGCTTAGAAATAGTATCGAAA	CCAGCTCTAGGCAGGCATAT	150	19-2B
D19Mit88	32.23	AACAGTGCAACTTTGGAGGC	TCATTGGAACTGTCTTAACAGTGC	148	19-3
D19Mit19	34.08	CCTGTGTCCATACAGGCTCA	ACCATATCAGGAAGCACCATG	138	19-19
D19Mit11	36.26	TCAAAGTCAAGGTGGGCAG	ACTTTCCAGATGTTGGGCAC	146	19-4
D19Mit1	50.32	AATCCTTGTTCACTCTATCAAGGC	CATGAAGAGTCCAGTAGAAACCTC	122	19-5
D19Mit71	56.28	ATGATTCCCGCAGTTTTGTC	TCTCAACTGTTATTCCTCAATAGCC	136	19-6

DXMit136	4.23	ACGGAAACACTCTTATGTGCG	ATTTTGATTACAGCATGTCCCC	182	X-136
DXMit48	25.51	ACCTCCCCCTGCATTACTCT	TTCTCCAGAATCCATGCTCC	105	X-48
DXMit62	34.6	GCAATTGTGATGTTGTAGTAAATATGG	ATAACTGAGGTCTGCGGGG	125	X-62
DXMit110	35.53	TGACATGAAGTATGTGTTCCTGC	TAGGCACATGTTCACATGGG	135	X-110
DXMit170	45.87	TGCAGGCACTAACAGTGAGG	TAGTTTCACTGTGCCATTGTATACA	115	X-170
DXMit179	53.17	TTTGATAGAGCCATGTTTGGC	CAGGCTAGCCTCAAACTCTCA	122	73.26
DXMit79	68.46	AGTCTGCCTTCTCTTTCTGTATCC	TGAAACTATTCCAACATTATTCTTGG	138	53.17
DXMit153	73.26	CAATCAAGCAGATGGAAGCA	AAGGACTGCCAAGAGGACAA	143	68.46

**Table:** (a) Category 1: markers that yielded the same size bands in all the strains

Marker	cM	Primer F	Primer R	Lab code
D2Mit74	103.43	CCAAGCTTGCAGTTTGTTAGC	AGGTGTTATTGAGCCCTGTATAGC	2-6A
D10Mit88	28.64	AAGATGAGAAGATAACATGTCAGGC	TTCTGAATTAAGTTCATCTGAACCC	10-2C
D11Mit226	5.64	AGGTGAACTCTTTTGAAGTTTGTG	AAAGGAGTGACTGAGAAAGACACC	11-1
D11Mit60	42.86	AGAGAGGCAAAAATTCCAAGC	CTTCCTGATGGTAGGATTTAGGC	11-60
D16Mit51	53.78	CCTCAGGTCAGTCAGGATTTAA	CCTGTTCACCCTCTCCACAT	16-5
DXMit57	16.24	AGTAGCAAGTAGACTCTCAAAGAGGG	TCTGGCATACATGGGCACT	X-57
DXMit81	20.59	GAGGAGCATCAACCTTCTCG	GAGGTGGGGAGAAACAGAGG	X-81
DXMit63	41.51	TTATAAATTAGTGTTACCACATGCAGC	AACATTTTTTTCCTAGCATGTGTG	X-63
DXMit186	76.75	ATCAATGCATAGTATTTGGGCC	AATTTGTCACTGCGGGTAGG	X-186

**Table:** (b) Category 2: markers that failed to amplify a PCR band in some strains

Marker	cM	Primer F	Primer R	Lab code
D1Mit375	23.18	TAAATCCATAGATGATAGATCAGTGTG	GTGGAAAAAAAACCTAAGACACC	1-2C
D1Mit76	33.31	ACAAAGGAAACTAAACAGACTCGG	CTCCCTCAAATACATCTTTGGC	1-3C
D1Mit308	50.78	GAGGCTATGAGTCAAATGGACC	TTTATGAGGTGCTGAGATGCA	1-5
D2Mit448	65.66	TACTGTTTGCATTTGAGTGCG	AAAAAGTAATGGTTGGGGCC	2-4B
D3Mit278	32.59	TCTAATATTGGAAAATGAATTTCTCTC	TATGCCCACATGCACACC	3-3P
D5Mit309	42.22	TAGAGCCTATTTCAAACCCCC	GTTGCATCCATAGCAAGCAA	5-4
D10Mit49	1.91	GGAATTTACACTGGAATACAACCC	GTGGGCATTTGCACTGTG	10-1
D11Mit168	78.74	CAGGGATTTGACTTTTAACCTCC	GAAATGGCTCCTACAACCTCC	11-168
D14Mit132	6.03	GAACAGCACCATCCACACAC	GTGGGGTTATATGCAGATACTCG	14-1
D15Mit147	46.85	GATGTGTGAAAAATTTTGTTTCTTG	GTCTCAAAGGAATAAGAAAGAGATGG	15-5
D17Mit3	34.9	GATCTTTTCTTATTCTGGTT	GCAAAGTCATGTACTCTGAG	17-3B
D17Mit39	45.64	CCTCTGAGGAGTAACCAAGCC	CACAGAGTTCTACCTCCAACCC	17-5
D18Mit48	51.89	TTGCACTCACAGGGCACAT	TCAGAGTTTCCAGAAGACACCA	18-6
D18Mit25	59.05	CTGGAAATAAAACCTGGGCA	TTTAGCCTAACTGAGTTCCAGACC	18-7
D19Mit31	8.84	CAATACAGAGTAATGATTGCCTGA	TTCACATTTTGGGATGCTCA	19-1B
D19Mit41	13.18	AGCCCTCCACCCAGTTTC	TCTGGGGAAAAAGGATGAGA	19-2
DXMit124a	4.25	AAGGAGAAGTAGAAGAAAGAAAAGAGG	GGTGTAGCCTCAAAAAAGATGG	X-124a
DXMit68	29.49	TCCTTTGGCCTCCTGCATAT	TGTTCTTACAATGAGCCTCATAGG	X-68
DXMit114	42.82	ATGGCATCCACAGTACCACA	GTAAAATCAATTTGTGAATAAGGAAGC	X-114
DXMit95	45.86	CTGTCAATCTAATTCTCTATGTCTGTG	CTTTCCTGGGTGGCAGTG	X-95
DXMit155	67.49	TGTGCACCACCACCATTC	AGGATCTGAGTGCCCAACC	X-155

**Table:** (c) Category 3: markers that amplified multiple PCR products in most (or all) strains

Marker	cM	Primer F	Primer R	Lab code
D11Mit86	32.13	TTGACATTGTGACAAAGACTTTCA	AAGGCATCATGAGGTTTTTAGTG	11-3
D12Mit118	44.93	CATCTTCAATAAAATGGAGATGTACA	CGCTTTCCCTTCATGTACTAGC	12-4
D13Mit107	50.2	CAACTGAGCCACATTCCTAGC	CAGCCACAGGATGATGAGG	13-4
DXMit98	72.38	GAGAGCAGGACTATGACTTG	ACACACCAGTTCGCACACAT	X-98
DXMit222	78.31	TTGGTTTGGGGTTTTTTTTG	ATTCCTGATAATGTCTTCTGGACA	X-222
